# UMP/CMPK Is Not the Critical Enzyme in the Metabolism of Pyrimidine Ribonucleotide and Activation of Deoxycytidine Analogs in Human RKO Cells

**DOI:** 10.1371/journal.pone.0019490

**Published:** 2011-05-03

**Authors:** Rong Hu, Wing Lam, Chih-Hung Hsu, Yung-Chi Cheng

**Affiliations:** 1 Department of Pharmacology, Yale University School of Medicine, New Haven, Connecticut, United States of America; 2 Department of Oncology, National Taiwan University Hospital, Taipei, Taiwan, Republic of China; German Cancer Research Center, Germany

## Abstract

**Background:**

Human UMP/CMP kinase was identified based on its enzymatic activity *in vitro*. The role of this protein is considered critical for the maintenance of pyrimidine nucleotide pool profile and for the metabolism of pyrimidine analogs in cells, based on the *in vitro* study of partially purified enzyme and recombinant protein. However, no detailed study has yet addressed the role of this protein in nucleotide metabolism in cells.

**Methodology/Principal Findings:**

Two stable cell lines in which UMP/CMP kinase (mRNA: AF087865, EC 2.7.4.14) can be either up-regulated or down-regulated were developed using Tet-On Gene Expression Systems. The amount and enzymatic activity of UMP/CMP kinase extracted from these two cell lines can be induced up by 500% or down by 95–98%. The ribonucleotides of endogenous pyrimidine as well as the metabolism of exogenous natural pyrimidine nucleosides and their analogs were not susceptible to the altered amount of UMP/CMP kinase in these two stable RKO cell lines. The level of incorporation of pyrimidine nucleoside analogs, such as gemcitabine (dFdC) and troxacitabine (L-OddC), into cellular DNA and their potency in inhibiting cell growth were not significantly altered by up-regulation or down-regulation of UMP/CMP kinase expression in cells.

**Conclusions/Significance:**

The UMP/CMP kinase (EC 2.7.4.14) expressed in RKO cells is not critical for the phosphorylation of (d)CMP and the maintenance of natural nucleotide pools. It also does not play an important role in the activation of dFdC and L-OddC. The increase by 500% or decrease by 95–98% in the levels of UMP/CMP kinase do not affect steady state levels of dFdC and L-OddC in RKO cells. Overall, the activity and possible mechanisms of recombinant UMP/CMP kinase expressed in the *in vitro* system can not be extended to that of UMP/CMP kinase expressed in a cell system or an *in vivo* system.

## Introduction

The enzymatic activity of UMP/CMP kinase has been considered to be essential for a variety of biological processes including RNA synthesis, DNA replication/repair, membrane phospholipids synthesis and nucleotide metabolism in cells. The human UMP/CMP kinase (mRNA: AF087865, EC 2.7.4.14) has been identified based on its enzymatic activity catalyzing the phosphorylation of CMP, UMP, dCMP and dUMP to their diphosphate nucleotides using partially purified protein from cellular extract as well as its recombinant protein [1]–[9]. The gene of this protein was found to be located on chromosome 1. (1p34.1-p33). It was assumed that this UMP/CMP kinase is responsible for the synthesis of CDP, UDP and dCDP as well as the diphosphates of deoxycytidine/deoxyuridine analog [2]–[9]. In the *in vitro* study, the efficiency of this protein could be affected by different conditions of the enzyme reaction, such as the concentration of DTT, Mg^+2^ and ATP *etc*. These could account for the difference of its properties among different laboratories. For the recombinant protein, its Km values for CMP, dCMP, UMP and dUMP are around 5–500 µM, 404–1600 µM, 50–1600 µM and 1300–5900 µM, respectively; the Kcat values for CMP, dCMP, UMP and dUMP are 1.7–248 s^−1^, 2.5–216 s^−1^, 0.6–140 s^−1^ and 0.1–7.2 s^−1^, respectively, as summarized in Table 1 [2], [5], [8], [10]. The physiological concentrations of CMP, dCMP, UMP and dUMP are around 34 µM, 0.3 µM, 184 µM and 2.2 µM, respectively, in cells [11]. The Michaelis-Menten kinetic parameters organized in Table 1, however, exhibit much higher Km values of this enzyme than the concentration of its natural substrates in cells. Based on its enzymatic characteristics, this enzyme would be inefficient in cells unless other associated protein(s) could improve its efficiency. If the amount of this protein is changed or the protein is modified such as up-regulation, down-regulation, phosphorylation, dephosphorlyation, glycosylation and methylation, it should affect the metabolism of nucleotides in cells. No detailed study currently has yet addressed the role of this protein on nucleotide metabolism in cells.

**Table 1:** Kinetic properties of recombinant human UMP/CMPK with ATP as phosphate donor.

Substrates	Km (µM)				Vmax (µmol/mg/min)				Kcat (s^−1^)			
CMP	500^a^	20^b^	15^c^	5∼29^d^	4.2^a^	350.0^b^	232.0^c^	513∼620.0^d^	1.7^a^	140.0^b^	92.0^c^	205∼248.0^d^
dCMP	1600	1000	513	404∼1388	602	213.5	65.0	288∼540..0	2.5	85.4	14.0	115∼216.0
UMP	1600	50	67	N.D	1.6	350.0	218.0	N.D	0.6	140.0	87.8	N.D
dUMP	5900	1300	N.D	N.D	0.1	17.9	N.D	N.D	0.1	7.2	N.D	N.D

The average values were summarized from the data reported by

a Van Rompay *et al*, 1999;

b Alexandre *et al*, 2007;

c Liou *et al*, 2002;

d Hsu *et al*, 2004 in the different enzyme assay condition.

Deoxycytidine analogs and 5-Fluorouracil (5-FU) are used in clinic for the treatment of certain tumors and viral infections. Those deoxycytidine analogs have to be phosphorylated stepwise to their triphosphate forms before they can be incorporated into cellular or viral DNA and/or RNA for anticancer and antiviral effects. UMP/CMP kinase activity should be pivotal for the cytotoxic effect of cytidine analogs in target cells because it is responsible for the phosphorylation of these cytidine analog monophosphates or 5-FU monophosphate to their diphosphate metabolites in anticancer and antiviral therapy [2], [5], [7], [9]. Gemcitabine (dFdC) and troxacitabine (L-OddC) are anticancer agents that are currently being used or under clinical trials for the treatment of cancer [12]. Although the configurations of dFdC and L-OddC are different, the first two steps in their metabolism appear to be the same. Both dFdC and L-OddC are phosphorylated by the cytoplasmic deoxycytidine kinase to their respective monophosphate metabolites, and the so-called UMP/CMP kinase *in vitro* can phosphorylate dFdC and L-OddC monophosphates to their diphosphate metabolites. Their *in vitro* Km values are 450–581 µM with Vmax 3.6–31 µmol/mg/min and 1037 µM with Vmax 0.63 µmol/mg/min, respectively [2], [5]. There are many enzymes involved in 5-FU metabolism, in which this UMP/CMP kinase was thought to play an important role in the activation of 5-FU to 5FUTP/5FdUTP and its incorporation into RNA and DNA [12].

Although the UMP/CMP kinase is suggested to be the enzyme responsible for the phosphorylation of (d)CMP and (d)UMP as well as some cytidine analog monophosphates, there is little information in cells to support the current dogma. The current knowledge indicated that the KEGG (Kyoto Encyclopedia of Genes and Genomes) metabolic pathway database has represented a network of interacting molecules as well as compensatory and regulatory pathways during pyrimidine metabolism in cells (http://www.genome.jp/kegg/pathway/map/map00240.html). However, a gene is not functionally identified until its phosphorylation target is identified or until the role on the biochemical pathway is identified [13].

In this study, we try to evaluate the impact of UMP/CMP kinase expressed in cells with respect to the metabolic and regulatory pathways of pyrimidine and its analogs. Two RKO cell lines were established in which the UMP/CMP kinase expression could be regulated either up or down by doxycycline. The intracellular ribonucleotide profile, pyrimidine nucleoside metabolism, dFdC and L-OddC metabolism, and their cytotoxicities were studied by altering the amount of UMP/CMP kinase in cells.

## Materials and Methods

### Chemicals and cell line

CMP, dCMP, ATP and 5-FU were purchased from Sigma. [5-^3^H(N)] Cyd, [5-^3^H(N)] dCyd, [5-^3^H] L-OddC, [5-^3^H] dFdC were purchased from Moravek Biochemicals. The double-stranded DNA oligonucleotide encoding the shRNA of UMP/CMP kinase with the following sequences were used: 5′-(+)CACCCGGGGAAATGGATCAGACAATGGCGAACCATTGTCTGATCCATTTCCC, 5′-(−)AAAAGGGAAATGGATCAGACAATGGTTCGCCATTGTCTGATCCATTTCCC (Invitrogen). RKO (human colorectal carcinoma) cells was a gift from Dr. Edward Chu's lab (Department of Internal Medicine, Yale University).

### Establishment of a stable cell line overexpressing UMP/CMP kinase

The inducible Tet-On UMP/CMP kinase cell line was established by using Tet-On Gene Expression System (Invitrogen). The procedures described in the user manual of the Tet-On Gene Expression System were followed. RKO cells were transfected using the regulator plasmid, pcDNA6/TR, which expressed tetracycline repressor (TR) protein. After 48 hr, the cells were grown in medium with 0.5 mg/mL blasticidin for selecting cells with TR expression. The selected cells with TR were further transfected using the pcDNA5/TO plasmids with the coding sequence (mRNA: AF087865) of human UMP/CMP kinase, the Tet-responsive element is between 5′- *BamH I* and 3′- *Xho I* sites of pcDNA5/TO vector (Invitrogen). To isolate cells with co-transfected constructs, selection medium with both blasticidin and hygromycin at 0.5 mg/mL was added after 48 hr transfection. The selected cells were used for the study. To induce the expression of UMP/CMP kinase, the cells grown in 5% serum-containing medium to 60% confluence were exposed to various concentrations of doxycycline for 72 hr. UMP/CMP kinase assays described below was used for evaluating the UMP/CMP kinase activity. The data were analyzed by GraphPad software.

### Establishment of the stable cell line underexpressing UMP/CMP kinase

To further explore the function of UMP/CMP kinase in cells, RNAi technology was used to down-regulate the expression of endogenous UMP/CMP kinase. BLOCK-iT™ Inducible H1 RNAi Entry Vector, pENTR/H1/TO, (Invitrogen) was used to generate a stable cell line that can be induced to express the short hairpin RNA (shRNA) against UMP/CMP kinase. A double-stranded DNA oligonucleotide generating a shRNA of UMP/CMP kinase was cloned into the H1/TO RNAi cassette located immediately downstream of the H1/TO pol III promoter. This H1/TO pol III promoter contains two TetO_2_ sites for tetracycline-regulated expression. The plasmid expressing shRNA was transfected into RKO cells with Lipofectamine 2000 (Invitrogen). To isolate stable cells with down-regulated UMP/CMP kinase effectively, the transfected cells were diluted 1∶60 into fresh medium for 48 hr, and then the selection medium was added. At all times, cells were grown in the presence of 0.75 mg/mL Zeocin for selection. To induce the expression of shRNA, the method used is similar to the one described above for overexpressing UMP/CMP kinase.

### Preparation of cell extract, UMP/CMP kinase Assays, Western blotting and immunostaining

All cell extracts were obtained as described [14]. The protein concentration of cell extracts was determined by using the Bio-Rad protein assay (Bio-Rad) and absorbance was read at 595 nm on kinetic microplate reader (Molecular Devices Corporation). The enzyme assays have been described previously [10]. In this study, we used the optimum condition for CMP and dCMP, respectively. The enzyme reaction was performed with 1 mM concentrations of substrates in the buffer containing 50 mM Tris-HCl, pH 7.5, 10 mM NaF, 25 mM NaCl, 2 mM dithiothreitol (DTT), 0.5 mM EDTA in which the ratios of ATP: Mg are 2∶2 mM for CMPK assay and 1∶2 mM for dCMPK assay. The supernatant part of cell lysates was loaded onto 12% SDS-PAGE gels, and then transferred onto pure nitrocellulose membrane (Bio-Rad) for Western blot assay. The blot was then incubated with the specific UMP/CMP kinase antibody [5], 3-phosphoglycerate kinase (PGK) recognized by PGK antibody [15] was used as the internal control. For the immunostaining of UMP/CMP kinase protein in cells, the procedure was carried out as previously described [16]. The UMP/CMP kinase antibody and Alexa Fluor 488 goat anti-rabbit IgG (Invitrogen) were used for the primary and secondary staining. The HSP27 protein was used as a control in co-localization studies using anti-HSP27 (Cell signaling) and Alexa Fluor 546 goat anti-mouse IgG (Invitrogen).

### Ribonucleotide pool assay

Endogenous nucleotide pool profiles were evaluated by increasing or decreasing the amounts of UMP/CMP kinase in cells. Cells with up-regulated or down-regulated UMP/CMP kinase were cultured in the 10% serum-containing medium with 0–2.0 ng/mL or 0–10.0 ng/mL doxcycline, respectively, for 72 hr. Cells were collected and treated with 15% trichloroacetic acid on ice for 10 min after being washed with ice-cold phosphate-buffered saline (PBS) containing 20 µM dipyridamole (Sigma) to obtain extracts. High pressure liquid chromatography (HPLC) with Partisil SAX column (Whatman) was used to assess the ribonucleotide profiles of the cellular supernatant extract treated with a 45∶55 ratio of trioctylamine and 1,1,2-trichlorotrifluroethane. The trichloroacetic acid insoluble pellet containing the nucleotide that has incorporated into the DNA was washed twice and resolubilized in Me_2_SO prior to evaluation on a Beckman LS5000TD scintillation counter. Results are based on the mean and standard deviation of at least three independent experiments.

### Metabolism of Cyd, dCyd, L-OddC and dFdC in cells overexpressing or underexpressing UMP/CMP kinase

The metabolism of radiolabeled Cyd, dCyd and their analogs was studied with the following procedures, which is a modification of the previously established protocol [16]. Briefly, the cells overexpressing and underexpressing UMP/CMP kinase were seeded at 5×10^5^ cells per culture dish, respectively. For the overexpression, doxycycline at 0, 0.4 and 2.0 ng/mL was added to the respective cells to induce UMP/CMP kinase; for underexpression, doxycycline 0, 1.0 and 10 ng/mL was added to respective cells to induce the shRNA of UMP/CMP kinase in order to down-regulate UMP/CMP kinase. After 72 hr, 1 µM [5-^3^H] Cyd (2.3 Ci/mmol), 1 µM [5-^3^H] dCyd (2.0 Ci/mmol), 2 µM [5-^3^H] L-OddC (0.4 Ci/mmol) or 2 µM [5-^3^H] dFdC (0.4 Ci/mmol), was added to cells induced by doxycycline, respectively, for indicated times. Parallel cultures were incubated with non-radiolabeled materials at same concentrations for calculating cell number. At the end of incubation with the radiolabeled materials, cells were harvested for making a crude cell extract. The cell supernatant and insoluble pellets then were further treated for HPLC assay and DNA incorporation analysis with the procedures described above.

### Growth inhibition assay

Growth inhibitory effects of the compounds were assessed by measuring the inhibition of cell proliferation. The experimental procedures have been described previously [17]. Briefly, RKO cells with overexpressing and underexpressing UMP/CMP kinase were pre-treated with or without doxycycline at different concentration for 72 hr. Then 10^4^ cells/mL in the logarithmic growth phase were seeded in 24-well plates and treated with dFdC (0–13 nM), L-OddC (0–600 nM) and 5-FU (0–20 µM). After maintaining the culture for 72 hr, the cell monolayer was then washed twice with PBS and stained with 0.5% methylene blue in 50% ethanol. The cell layer was washed with water, dried, and solubilized in 1% N-lauroyl-sarcosine (Sigma). The protein concentration was determined by reading *A*_595_ nm on an automated microplate reader (Bio-Tek Instrument, Inc.). The potency of the compounds examined for cell growth inhibition was then determined by comparing the treated wells with the untreated control wells.

## Results

### Evaluation of cells with up-regulated and down-regulated UMP/CMP kinase systems

**Figure 1:**
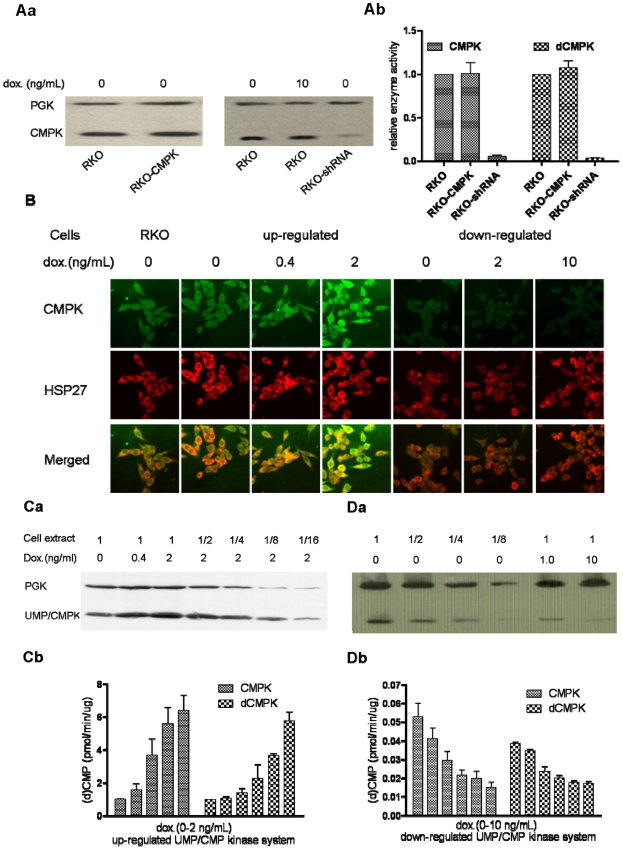
Protein amount and enzymatic activity of UMP/CMPK in cellular extract from RKO cells. Doxycycline induces the overexpression or underexpression of UMP/CMP kinase. Specific antibodies of UMP/CMP kinase and PGK were used to detect the proteins of UMP/CMP kinase and PGK, respectively, for Western Blot. ***Aa.*** parental RKO cells with or without doxycycline induction. RKO-CMPK (cells with up-regulated UMP/CMP kinase system) and RKO-shRNA (cells with down-regulated UMP/CMP kinase system) without doxycycline induction. ***Ab.*** (d)CMP kinase assays for parental RKO cells, RKO-CMPK and RKO-shRNA cells without doxycycline induction. ***B.*** confocal microscopy images of cells induced by different concentration of doxycycline, which show the localization of UMP/CMP kinase (green fluorescence) and HSP27 (red fluorescence). Parental RKO cells were used as control cells. ***C.*** overexpression of UMP/CMP kinase in RKO cells. ***a.*** expression of UMP/CMP kinase level was detected by Western blotting. PGK is used as the internal control. ***b.*** enzyme activity of UMP/CMP kinase using CMP and dCMP as substrates. ***D.*** underexpression of UMP/CMP kinase in RKO cells. ***a.*** expression of UMP/CMP kinase level was detected by Western blotting. PGK is used as the internal control. ***b.*** enzyme activity of UMP/CMP kinase using CMP and dCMP as substrates.

To determine whether UMP/CMP kinase affects the nucleotide metabolism and the phosphorylation of deoxycytidine analogs, we have established two stable cell lines in which UMP/CMP kinase can be up-regulated and down-regulated, respectively. Under non-inducted condition, the protein levels and enzymes activities of UMP/CMP kinase in cells with the up-regulated system are comparable to that of control cells. However, we observed that the protein expression of UMP/CMP kinase dropped down and the enzyme activities reduced conversion by 93% for CMP and 96% for dCMP in cells with shRNA-mediated knockdown under non-induced condition. This could be due to the leakage of the H1 promoter which has been reported by others [18]. We also found out that the longer time in culture, the more leakage occurs. No change in protein amount was found in parental RKO cells with/without doxycycline treatment. (Fig. 1Aa). The digital images of cells demonstrated the subcellular localization of UMP/CMP kinase. Although the amount of enzyme can not be exactly measured by the technique of confocal microscope, the expression of UMP/CMP kinase was clearly overexpressed in cells with the up-regulated UMP/CMP kinase system after doxycycline induction. In down-regulated UMP/CMP kinase cells, the images show that the expression of UMP/CMP kinase has reached a base line level as compared with control cells (Fig. 1B).

### Endogenous ribonucleotide pool profiles and metabolism of exogenous Cyd and dCyd are not affected by overexpressing or underexpressing UMP/CMP kinase in cells

**Figure 2:**
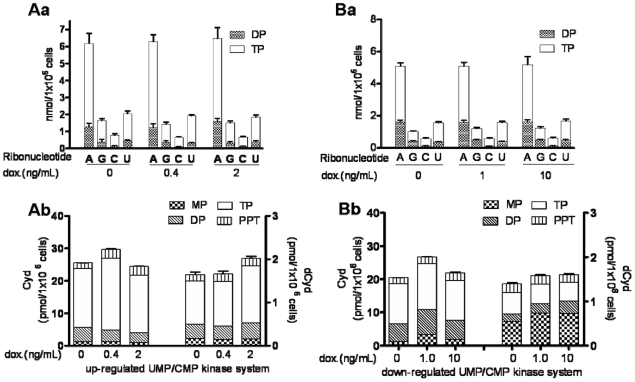
Evaluation of endogenous ribonucleotide pool profile and metabolism of exogenous Cyd and dCyd in RKO cells overexpressing or underexpressing UMP/CMP kinase. ***Aa.*** the content of ribonucleoside di- and triphosphates in cells overexpressing UMP/CMP kinase. ***Ab.*** the amounts of Cyd and dCyd metabolites as well as the amounts of their incorporation into DNA (PPT) in cells overexpressing UMP/CMP kinase. ***Ba.*** the content of ribonucleoside di- and triphosphates in cells underexpressing UMP/CMP kinase. ***Bb.*** the amounts of metabolites of Cyd and dCyd as well as the amounts of their incorporation into DNA (PPT) in cells underexpressing UMP/CMP kinase.

The cell lines with up-regulated or down-regulated UMP/CMP kinase system induced by doxycycline were used to evaluate the impact of UMP/CMP kinase on endogenous pools of ribonucleoside diphosphate and triphosphate in cells. The UMP/CMP kinase induced by doxcycline was monitored by enzyme assay and Western blot analysis. In the *in vitro* assays, as shown in Fig. 1C,D, different protein levels and activities of UMP/CMP kinase were observed in non-induced cells (control group) between both cell lines. The enzymatic activities (Fig. 1 Cb,Db) and the amount (Fig. 1 Ca,Da) of UMP/CMP kinase in the cell extracts from the two cell lines are dependent on the different concentrations of doxycycline and show a maximum alteration of around 500% over no doxycycline induction. In cells with 500% UMP/CMP kinase increase or in cells with total 95–98% decrease, *i.e.* leaky 93–96% plus inducible 2% (Fig. 1), there is no alteration on the profiles of the purine and pyrimidine ribonucleotides (Fig. 2 Aa,Ba). The metabolism of exogenous Cyd and dCyd were examined in these two cell lines. The amount of diphosphate or triphosphate metabolite of Cyd or dCyd is not affected by altering the amount of UMP/CMP kinase in cells (Fig. 2 Ab,Bb). In the system down-regulating UMP/CMP kinase, the level of dCyd monophosphate in non-induced cells is higher compared to that in the system up-regulating UMP/CMP kinase. It can not be excluded that the shRNA nonspecifically targets another protein. No change in dCyd metabolites, however, was found after doxycycline induction as compared with non-induced cells. Therefore, overexpression and underexpression of UMP/CMP kinase do not have a major impact on the incorporation of Cyd and dCyd into RNA or DNA (Fig. 2 Ab,Bb).

### No effect on the metabolism of dFdC and L-OddC in up-regulated and down-regulated UMP/CMP kinase cells

**Figure 3:**
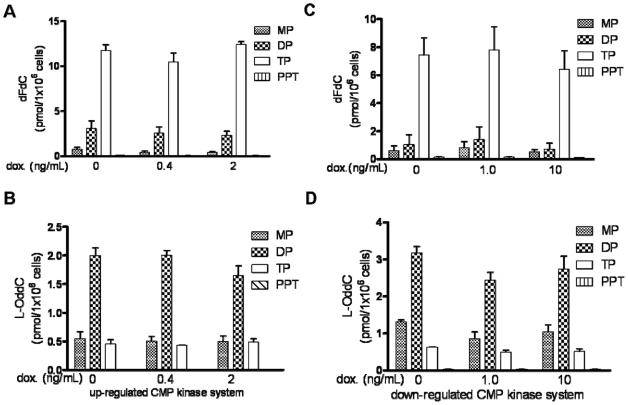
The metabolism of dFdC and L-OddC in cells overexpressing or underexpressing UMP/CMP kinase. ***A.*** the content of dFdC mono-, di- and triphosphate in cells overexpressing UMP/CMP kinase. ***B.*** the content of L-OddC metabolites in cells overexpressing UMP/CMP kinase. ***C.*** the content of dFdC metabolites in cells underexpressing UMP/CMP kinase. ***D.*** the content of L-OddC metabolites in cells underexpressing UMP/CMP kinase.

The effect of overexpressing or underexpressing UMP/CMP kinase on the metabolism of dFdC and L-OddC was examined in cells. Both sets of inducible cell lines were cultured in medium with different concentrations of doxycycline for 72 hr, then treated with 2 µM dFdC or 2 µM L-OddC, respectively, for 6 hr. In cells with 500% increase or total 95–98% decrease in UMP/CMP kinase expression, the dFdC metabolite levels remain unaffected. The ratios of dFdC monophosphate to its diphosphate and triphosphate are also similar. The amount of dFdC triphosphate incorporated into the DNA remained constant in comparison with the control group in both cells overexpressing and underexpressing UMP/CMP kinase (Fig. 3 A,C). The same results are also observed for metabolism of L-OddC in both inducible cell lines (Fig. 3 B,D).

### Overexpression and underexpression of UMP/CMP kinase do not influence the growth-inhibition activity of dFdC, L-OddC and 5-FU in RKO cells

**Figure 4:**
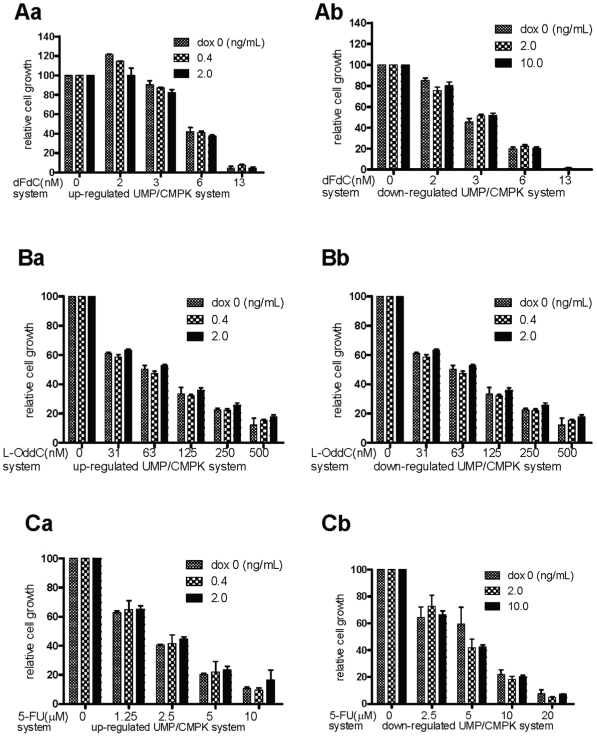
Growth-inhibition activity of deoxycytidine analogs and 5-FU in cells up-regulating or dow-nregulating UMP/CMP kinase expression. ***Aa,Ba,Ca.*** growth-inhibition activity of dFdC, L-OddC and 5-FU in cells up-regulating UMP/CMP kinase with different concentration of doxycycline. ***Ab,Bb,Cb.*** growth-inhibition activity of dFdC, L-OddC and 5-FU in cells down-regulating UMP/CMP kinase with different concentration of doxycycline.

The objective of our study was to evaluate the efficiency of intracellular UMP/MP kinase to facilitate the cytotoxic activities of dFdC, L-OddC and 5-FU and the correlation of triphosphates with the pharmacological activities of these drugs. For the up-regulated UMP/CMP kinase cells in the presence of different concentration of doxycycline (0, 0.4 and 2.0 ng/mL), compared with the control group (doxycycline 0 ng/mL), results of 72 hr growth-inhibition showed that the differential effects of doxycycline used at 0.4 and 2.0 ng/mL dosages have not significant change in all drug treatments. Two-way ANOVA analysis shows that their P values are 0.849 and 0.091 for dFdC. For L-OddC, the P values are 0.598 and 0.092. The P values are 0.760 and 0.069 for 5-FU. For the down-regulated UMP/CMP kinase cells induced by doxycycline, the various dosages (2.0, 10.0 ng/mL) of doxycycline have no significant effect for all drug treatments as compared with control group. The P values of dFdC are 0.919 and 0.652. The P value of L-OddC are 0.391 and 0.392. The P values of 5-FU are 0.379 and 0.305. As shown in Fig. 4, the inhibitory potency on cell growth is not altered by change in amount of UMP/CMP kinase in cells treated with drugs for 72 hr. The results are consistent with the data shown in Fig. 3.

## Discussion

Human UMP/CMP kinase was identified based on its enzymatic activity *in vitro*. The majority of studies on mammalian UMP/CMP kinase have been performed with partially purified enzymes from different cells and tissues [2]. The cDNA of human UMP/CMP kinase has been cloned, expressed and purified. The recombinant protein has been used to study the phosphorylation of both natural (d)CMP and their analogs. The crystal structure further explained its kinetic properties for different substrates [19]. Its major biochemical characteristics based on enzymatic activities in the *in vitro* system were summarized in Table 1. We have established inducible UMP/CMP kinase cell lines as cell models in an attempt to understand the active pathways of nucleotide metabolism. We have focused on the metabolism of endogenous and exogenous (d)cytidine as well as the activation of their nucleoside analogs in cells. Our results indicated that either the level of UMP/CMP kinase can be increased by 500% or decreased by 95–98%. However, the natural triphosphate levels of adenosine, guanosine, uridine and cytidine are not altered and the metabolism of exogenous cytidine and deoxycytidine is not changed in these cell lines. The differences of enzyme activities between *in vitro* system and in cell system could be due to: (1) the characteristic of recombinant UMP/CMP kinase protein expressed in *E. coli* may be different from that of the protein expressed in cells. In the cell system, there is a major contribution of post-translational modifications in cells to the expressed protein for efficient secretion and stability. These protein modifications include correct folding and aggregation, oxidation of methionine, deamidation of asparagines and glutamine, variable glycosylation, and proteolysis. In contrast, the recombinant proteins expressed in bacteria undergo simple modifications. [20]. (2) The pathways of nucleoside metabolism consist of complex molecular interaction networks involving many enzymes of phosphorylation/dephosphorylation, molecular bindings and proteolysis, in addition to the control of gene expression. On the KEGG metabolic pathway map, the metabolism of (d)CMP currently consists of 104 functionally related enzymes (Supplementary Fig. S1). In cells with overexpressed UMP/CMP kinase, the feedback regulation of different enzymes and their products would balance the impact of UMP/CMP kinase once it dominates the conversion from (d)CMP to (d)CDP in the presence of doxycycline. In cells with underexpressed UMP/CMP kinase, the conversion of (d)cytidine could have many alternative pathways instead. Thus, the results demonstrated that this UMP/CMP kinase, either expressed in abundance or reduced to low levels, does not play a role on the metabolism of pyrimidine or the maintenance of pyrimidine pool profiles in RKO cells. Since the cellular pools are not sensitive to UMP/CMP kinase, the up-regulation or down-regulation of UMP/CMP kinase might have triggerd compensatory mechanisms. These results indicated that the activities and possible mechanisms of protein expressed in the *in vitro* system can not be extended to that of UMP/CMP kinase protein expressed in a cell system or an *in vivo* system.

Our observations also argue that this UMP/CMP kinase interferes with the phosphorylation of dFdC and L-OddC. The cytotoxycity data also support the metabolism observation and show that the UMP/CMP kinase might not be critical for the maintenance of steady state levels of dFdC, L-OddC and 5-FU in cells. Interestingly, cell survival assay using a colony formation reported by Liou *et. al.* [21] demonstrated that up-regulation and down-regulation of UMP/CMP kinase protein lead to a change in the cellular sensitivity to dFdC due to the increased formation of total amount of diphosphate and triphosphate metabolites in HeLa S3 and HCT-8 cells. However, one may assume that there is another compensatory pathway to phosphorylate these nucleoside analogs stepwise to their triphosphates. It is important to understand the mechanism of the numerous anticancer and antiviral deoxycytidine analogs which must be activated to their respective triphosphates. In addition, molecular alterations also may have the potential to affect cell survival after analogs treatment. The dFdC in 100 nM concentration can effectively induce the expression of *p53* and *Bax*, and then lead to apoptosis in RKO cells [22]; human apurinic/apyrimidinic endonuclease (APE-1) exonuclease activity plays an important role in the cytotoxicity of L-OddC [23]. Russo P *et. al.* demonstrated that the cytotoxicity of 5-FU (0.01–10 uM) correlates with the status of *p53* gene and *Ras* gene in ten human colon cancer cell lines [24]. Nucleoside analogs-mediated apoptosis, cytotoxicity and cell cycle progression are influenced by p53 status, Ras, and APE-1 level in cells. The up-regulation or down-regulation of UMP/CMP kinase might be associated with these pathways. These observations may explain the discrepancies between cytotoxicity and cell survival assay in which cells were exposed to 5-FU (1–100 uM) demonstrated by Humeniuk *et. al.* [25], [26]. Furthermore, they also suggested there could be a small impact on 5-FU sensitivity once the amount of UMP/CMP kinase changes in cells.

In summary, the UMP/CMP kinase (EC 2.7.4.14) is not critical for the phosphorylation of CMP, dCMP and maintenance of natural nucleotide pools in cells. It also does not play an important role in the activation of dFdC and L-OddC. The increase by 500% or decrease by 95–98% in its cellular levels does not affect steady state levels of dFdC and L-OddC in RKO cells. Our present results are not consistent with previous reference reports since we expressed the protein in cell systems instead of in *in vitro* systems. Overall, the activity and possible mechanisms of recombinant UMP/CMP kinase expressed in the *in vitro* systems can not be extended to that of UMP/CMP kinase expressed in a cell system or an *in vivo* system.

## Supporting Information

Figure S1 **Pyrimidine metabolism pathway.** This pathway depicted by the KEGG was released on Oct. 19, 2010 at http://genome.jp/kegg/pathway/map00240.html. The enzymes are shown in boxes with the EC numbers inside. The circled box represents the UMP/CMP kinase (EC 2.7.4.14), the recombinant version of which was used in our study.
(TIF)
