# Supplementary figures and images for: UMP/CMPK Is Not the Critical Enzyme in the Metabolism of Pyrimidine Ribonucleotide and Activation of Deoxycytidine Analogs in Human RKO Cells
Source: PLoS One. 2011 May 3;6(5):e19490. doi: 10.1371/journal.pone.0019490 (PMC3086915; doi:10.1371/journal.pone.0019490)

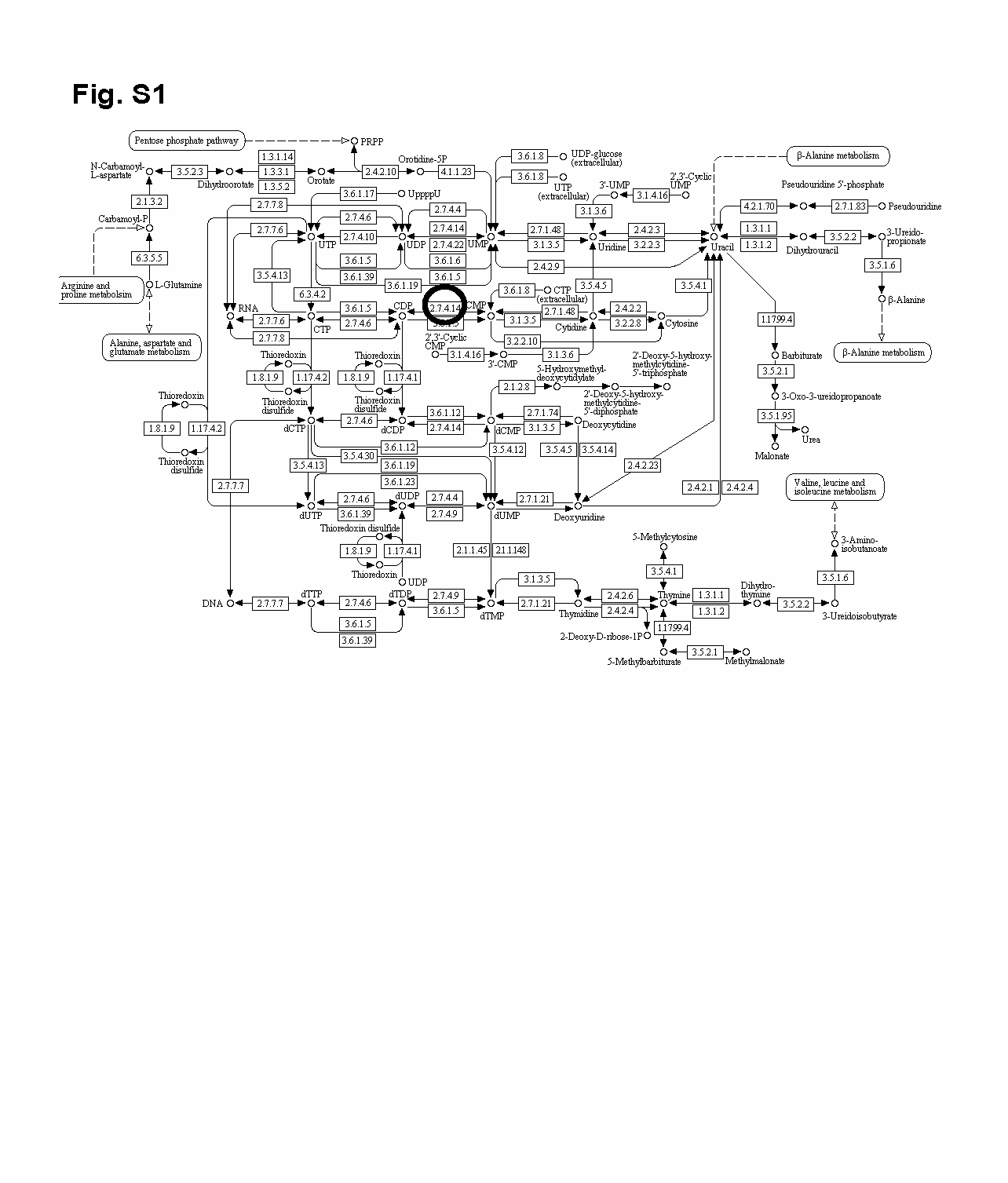

Supplement: Figure S1 — Pyrimidine metabolism pathway. This pathway depicted by the KEGG was released on Oct. 19, 2010 at http://genome.jp/kegg/pathway/map00240.html. The enzymes are shown in boxes with the EC numbers inside. The circled box represents the UMP/CMP kinase (EC 2.7.4.14), the recombinant version of which was used in our study. (TIF) [file pone.0019490.s001.tif]
